# Seasonal Trends in Hospitalization of Attempted Suicide and Self-Inflicted Injury in United States Adults

**DOI:** 10.7759/cureus.10830

**Published:** 2020-10-06

**Authors:** Adeolu O Oladunjoye, Olubunmi O Oladunjoye, Oluwatosin A Ayeni, Oluwatoyin Olubiyi, Anna Fuchs, John Gurski, Maria Ruiza Yee, Eduardo D Espiridion

**Affiliations:** 1 Psychiatry, Reading Hospital Tower Health, West Reading, USA; 2 Medical Critical Care, Boston Children's Hospital, Boston, USA; 3 Internal Medicine, Reading Hospital Tower Health, West Reading, USA; 4 Non-Communicable Diseases, Wits Health Consortium, Johannesburg, ZAF; 5 Public Health, Philadelphia Department of Health, Philadelphia, USA; 6 Medicine, Drexel University College of Medicine, West Reading, USA; 7 Psychiatry, Philadelphia College of Osteopathic Medicine, Philadelphia, USA; 8 Psychiatry, Drexel University College of Medicine, Philadelphia, USA; 9 Psychiatry, West Virginia School of Osteopathic Medicine, Lewisburg, USA; 10 Psychiatry, West Virginia University School of Medicine, Martinsburg, USA

**Keywords:** hospitalization, self-inflicted injury, self-harm, suicide prevention, suicide

## Abstract

Introduction

Suicide is the 10th leading cause of death in the United States (US) and the prevalence continues to increase. It is estimated that there is an average of 25 attempted suicides for every suicide death in the US, and the economic burden of suicide and attempted suicide is high. Identification of those at risk for suicide and attempted suicide can help with early and prompt intervention. Studies in Europe and Asia have shown that there is a relationship between seasonal patterns and suicidal risk. However, little is known about seasonal patterns of suicidal attempts in the US. Therefore, our study aimed to assess seasonal patterns by days of the week and months of the year in the US.

Methods

Hospitalized adult patients with suicide attempts and self-inflicted injury were identified using the discharge data from the National Inpatient Sample (NIS) from January 1, 2010 to December 31, 2014. We looked at the seasonal trends of patients with attempted suicide and self-inflicted injury by weekday vs weekend and month of the year over the five-year study period. We also assessed two groups, male and female with attempted suicide and compared trends and contributing risk factors over the study period using Student’s t-test and chi-square test.

Results

A total of 249,845 patients with attempted suicide and self-inflicted injury were reported during the study period with a prevalence rate increase of 15%, among which 70% were males, 65.5% white and 38.8% were age 40-64 years. An overall prevalence rate of about 168-200 per 100,000 hospitalizations was reported. There was a higher admission rate on weekends as compared to weekdays (190-300 vs 150-178 per 100,000 hospitalizations). Attempted suicide and self-inflicted injury admissions peaked during the months of July and August with a peak period range of 200-230 per 100,000 hospitalizations in a year.

Conclusion

The prevalence of attempted suicide is steadily rising. Awareness of the seasonal and epidemiological trends of attempted suicide and self-inflicted injury is a very important step towards developing effective strategies to prevent suicide and attempted suicide.

## Introduction

According to the Centers for Disease Control and Prevention (CDC), suicide is the 10th leading cause of death in the United States (US) [1]. Between 2005 and 2014 the CDC reported a rise in the cases of suicide death by 31%, an increase from 32,637 to 42,773 [2]. This number continues to rise with the number of attempted suicides increasing as well. It is estimated that there is an average of 25 attempts for every suicide death in the US [3]. In 2018, 1.4 million adult Americans attempted suicide and 48,000 actually died by suicide [1,4].

One major way to prevent suicide is the identification and treatment of those who are at high risk such as those with a previous suicide attempt. Attempted suicide is a major source of concern, particularly due to its link with future attempts and suicide completion [5]. Approximately 18% of those who attempt suicide make a second attempt during the following year [6]. A Swedish study found that the rate of suicide among those with an attempt the previous year was 100-fold higher than the suicide rate in the age- and sex-matched control group [7]. During the first year after a suicide attempt, the risk of completed suicide in men is 0.8 to 3.0% while in women, it is 0.3 to 1.9% [7-9].

The economic burden of suicide and attempted suicide in the US is about 70 billion dollars annually in lifetime medical and work-loss costs alone [1]. Many of those who attempt suicide have mood disorders or substance use disorders [6]. Also, those who survive attempted suicide may suffer from consequences such as brain injury and broken bones. Population-based surveillance that assesses patterns can help to identify ways of reducing suicidal behaviors. In Europe and Asia, there is an increasing number of studies that suggest that there is a relationship between seasonal patterns and suicidal risk [10-12]. Epidemiological studies on seasonal variation have been studied since the early 20th century. In his book published in 1951, Durkheim found that lower rates of suicide occur in the second half of the year [13]. He also found a higher rate on the first day of each week. Other studies in the 21st century found that the first month of the year and the first days of the week had the highest rates [14,15]. Some other studies have found seasonal patterns with peaks in the summer or fall [10,16]. However, little is known about seasonal patterns of suicidal attempts in the US and how this seasonal pattern has changed over the years. We assessed seasonal patterns by days of the week and months of the year using the National Inpatient Sample (NIS) database, the largest all-payer inpatient database in the US.

## Materials and methods

Study design and data sources

The study was based on the discharge data from the NIS database administered by the Agency for Healthcare Research and Quality - a part of the Health care Cost and Utilization Project (HCUP) [17]. The NIS is the largest all-payer publicly available inpatient care database made up of 20% sample of US hospitalizations, with more than 40 states in the US, the weighted estimate of which represents >95% of all hospitalized US population. Each year NIS has about 7 million hospitalization records (weighted to 35 million hospitalizations) [17]. We analyzed all adult admissions (18 years and above) from January 1, 2010 to December 31, 2014.

International Classification of Diseases Ninth Revision Clinical Modification (ICD-9-CM) was derived from 25-30 diagnoses columns that were used to identify the study population. Quality control procedures performed by the HCUP have demonstrated reliability and accuracy, mainly when data contain the principal diagnosis. Since the database is de-identified and publicly available, ethical clearance or Institutional Review Board approval was not necessary.

Study population and characterization of variables

**Figure 1:**
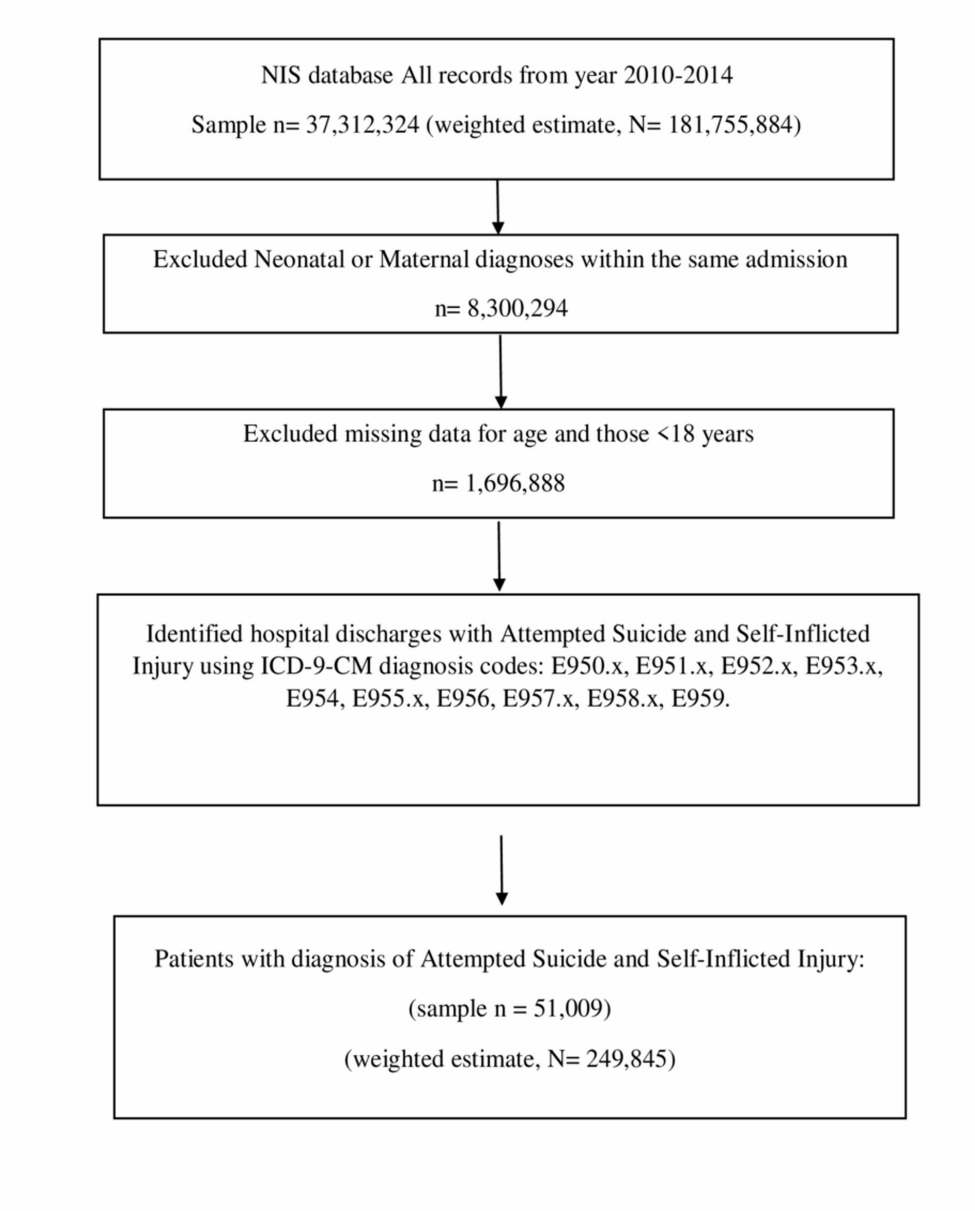
Flowchart for attempted suicide and self-inflicted injury hospitalizations in the United States n, sample number; N, weighted average estimate; ICD-9, International Classification of Diseases 9; NIS, National Inpatient Sample

The ICD-9-CM procedure and diagnosis codes were used to identify the diagnosis of interest in the NIS database. We identified ICD-9-CM diagnosis codes for attempted suicide and self-inflicted injury with its subtypes of attempted suicide using E950-E959 diagnostic codes (Figure 1).

We also looked at various risk factors that might contribute to suicide attempts and used ICD-9 codes to identify common mental health disorders and substance abuse disorders. We looked at the seasonal trend of patients with attempted suicide and self-inflicted injury by weekday vs weekend and months of the year over the five-year study period. We also assessed two groups, male and female, and compared their trends and risk factors that may contribute to suicidality over the study period.

Patient demographics and comorbidities

Patient-level characteristics from the database included age (sub-divided into 18-24, 25-39, 40-64, and 65+ years), race (white, black, and others), primary payer (government, private, self-pay, and others), zip code-based annual median household income (divided into four quartiles), regions of the US (northeast, south, mid-west/north-central, and west). Clinical characteristics were derived from the database for patients who had mental health disorders (including anxiety, depression, and psychosis) and substance use disorders (including alcohol, marijuana, sedative-hypnotic, cocaine, stimulant, and hallucinogen). 

Statistical analysis

Stata version 15.0 (StataCorp., College Station, TX, USA) was used for all statistical analyses. Categorical variables were reported as numbers and percentages while continuous variables were presented as mean and standard deviation. Differences between male and female groups were compared using Student’s t-test and chi-square test when appropriate. Univariate and multivariate analysis using logistic regression was done to determine the predictors of attempted suicide and self-inflicted injury. We used a P-value of <0.05 and 95% confidence interval (CI) to show statistical significance. Linear models were used to derive trend analysis using the Joinpoint regression analysis statistical software to derive annual percentage change (APC). The Joinpoint software takes trend data and based on the maximum number of joinpoints supplied by the user, fits the data into segments, enabling the users to assess if the apparent change in trend is statistically significant [18]. All analyses were performed with strata and weight to account for the complex clustered sampling methodology.

## Results

Descriptive characteristics of hospitalized patients with attempted suicide and self-inflicted injury

**Figure 2:**
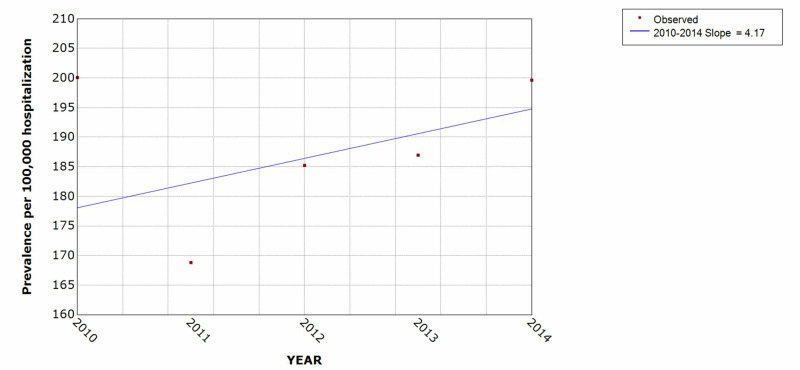
Trend of attempted suicide and self-inflicted injury hospitalization over the five-year period * indicates that the slope is significantly different from zero at the alpha = 0.05 level. Final selected model: 0 Joinpoints

We studied a total of 249,845 patients with attempted suicide and self-inflicted injury during the five-year study period of January 1, 2010 to December 31, 2014, among which 176,133 (70%) were males. The prevalence of patients with attempted suicide and self-inflicted injury increased from 2011 to 2014 by 15% over the five-year period (Figure 2).

The patients with attempted suicide and self-inflicted injury comprised 65.5% white and 38.8% age 40-64 years old. The overall mean age was 48.8±0.2 years. The demographic and clinical characteristics of attempted suicide and self-inflicted injury were compared between gender (Table 1). The proportion of patients with attempted suicide and self-inflicted injury was highest among those in the lowest income quartile (32.5%), those on government insurance (41.2%), those from southern hospitals (39.8%), and those in the urban teaching hospitals (71.6%).

**Table 1:** Baseline characteristics of hospitalized patients with attempted suicide and self-inflicted injury N: Weighted average; n: sample number; SE: Standard error; %: percentage

Name	Overall (n= 51,009) (N= 249,845)	Male (n=35,707) (N= 176,133)	Female (n=15,302) (N=73,712)	P-Value
Mean Age (±SE)	48.8±0.2	46.1±0.2	55.2±0.3	<0.0001
Age, years				
18-24	14.2	16.1	9.9	
25-39	23.0	25.6	16.8	
40-64	38.8	39.7	36.8	
≥ 65	24.0	18.6	36.5	<0.0001
Race, %				
White	65.5	62.5	72.7	
Black	15.5	17.0	11.8	
Others	19.0	20.5	15.5	<0.0001
Mental health disorders, %				
Yes	10.5	8.1	16.2	
No	89.5	91.9	83.8	<0.0001
Substance use disorder, %				
Yes	16.4	18.8	10.5	
No	83.6	81.2	89.5	<0.0001
Depression, %	8.2	6.2	12.9	<0.0001
Alcohol Abuse, %	11.8	13.8	7.0	<0.0001
Obesity, %	6.5	5.0	10.0	<0.0001
Income, %				
First quartile	32.5	33.5	30.2	
Second quartile	26.2	26.2	26.2	
Third quartile	23.3	23.1	23.6	
Fourth quartile	18.0	17.3	20.0	<0.0001
Insurance, %				
Government	41.2	36.4	52.7	
Private	32.3	32.0	32.9	
Self- Pay	14.4	17.0	8.4	
Others	12.1	14.6	6.0	<0.0001
Region, %				
North East	16.5	16.6	16.5	
Mid-West/North Central	20.2	19.9	20.9	
South	39.8	39.4	40.5	
West	23.5	24.1	22.2	0.0096
Hospital Teaching Status, %				
Rural	5.8	5.3	7.0	
Urban non-teaching	22.6	21.0	26.3	
Urban teaching	71.6	73.7	66.7	<0.0001

Trends of patients with attempted suicide and self-inflicted injury

**Figure 3:**
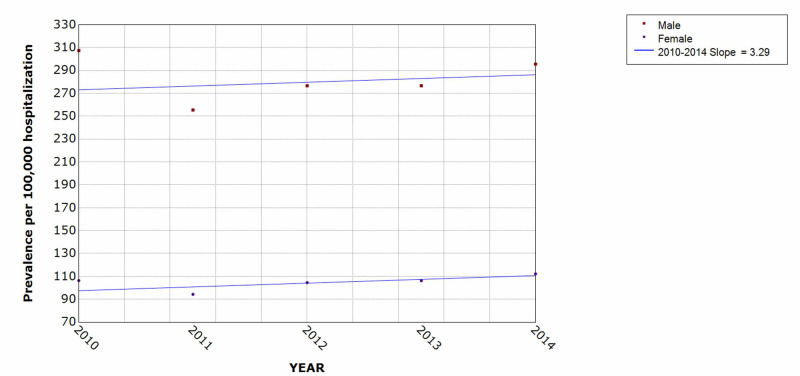
Trend of attempted suicide and self-inflicted injury hospitalization by gender over the five-year period * indicates that the slope is significantly different from zero at the alpha = 0.05 level. Final selected model: 0 Joinpoints

**Figure 4:**
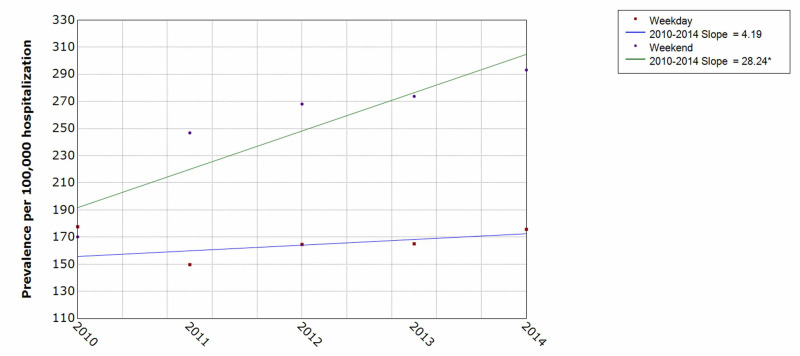
Weekdays vs weekends trends of attempted suicide and self-inflicted injury hospitalization over the five-year period * indicates that the slope is significantly different from zero at the alpha = 0.05 level. Final selected model: 0 Joinpoints, vs: versus

Patients with attempted suicide and self-inflicted injury showed an overall prevalence rate of about 168-200 per 100,000 hospitalizations. The trend in male vs female populations showed that males had a higher prevalence with 255-295 per 100,000 hospitalizations compared to females with 95-112 per 100,000 hospitalizations (Figure 3). There was also a higher prevalence rate on weekends compared with weekdays (190-300 vs 150-178 per 100,000) (Figure 4).

**Figure 5:**
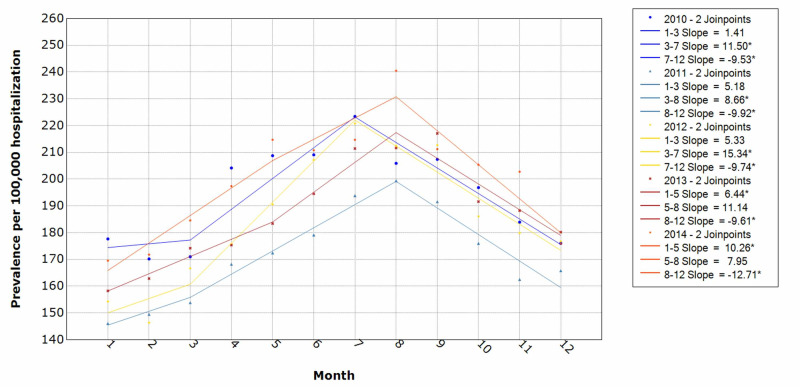
Yearly trends of attempted suicide and self-inflicted injury hospitalization * indicates that the slope is significantly different from zero at the alpha = 0.05 level. Final selected model: 0 Joinpoints

Comparing prevalence by month over the five-year study period, July and August were the peak periods of patients who had attempted suicide and self-inflicted injury. Peak periods ranged with a prevalence of 200-230 per 100,000 hospitalizations per year. The trend pattern from January also showed a steady rise until March with a steep rise from March till it peaked around July/August. Prevalence declined throughout the remainder of the year (Figure 5).

Predictors of patients with attempted suicide and self-inflicted injury

After adjusting for other variables, multivariate logistic regression (odds ratio (OR) (95% CI), p<0.001) determined the following factors were associated with increased odds of attempted suicide and self-inflicted injury: alcohol abuse (OR 1.64 (1.55-1.73)); those on private insurance (OR 1.37 (1.32-1.44)); those who self-pay (OR 2.04 (1.93-2.17)) and those on other types of insurance (OR 2.89 (2.74-3.05)) (Table 2).

**Table 2:** Factors associated with hospitalized patients with attempted suicide and self-inflicted injury SE: Standard error, OR: Odds ratio, Ref: Reference

Name	Univariate analysis (Crude OR)	P-Value	Multivariate analysis (Adjusted OR)	P-Value
Mean Age (±SE)	0.96 (0.96-0.97)	<0.0001		
Age, years				
18-24	Ref			
25-39	0.54 (0.52-0.56)	<0.0001	0.54 (0.52-0.56)	<0.0001
40-64	0.23 (0.22-0.24)	<0.0001	0.25 (0.24-0.26)	<0.0001
≥ 65	0.12 (0.11-0.12)	<0.0001	0.16 (0.15-0.17)	<0.0001
Race				
White	Ref			
Black	1.12 (1.05-1.20)	0.001	0.84 (0.79-0.90)	<0.0001
Other	1.44 (1.34-1.57)	<0.0001	1.03 (0.96-1.11)	0.365
Mental health disorder				
No	Ref			
Yes	0.60 (0.58-0.63)	<0.0001	0.55 (0.54-0.58)	<0.0001
Substance use disorder				
No	Ref			
Yes	1.84 (1.77-1.90)	<0.0001	0.94 (0.89-0.99)	0.046
Depression	0.59 (0.57-0.61)	<0.0001	0.66 (0.63-0.69)	<0.0001
Alcohol Abuse	1.95 (1.88-2.03)	<0.0001	1.64 (1.55-1.73)	<0.0001
Obesity	0.49 (0.47-0.51)	<0.0001	0.52 (0.50-0.55)	<0.0001
Income				
First quartile	Ref			
Second quartile	0.93 (0.89-0.97)	<0.0001	0.95 (0.91-0.99)	0.010
Third quartile	0.90 (0.85-0.95)	<0.0001	0.91 (0.86-0.96)	0.001
Fourth quartile	0.83 (0.77-0.89)	<0.0001	0.84 (0.78-0.91)	<0.0001
Insurance				
Government	Ref			
Private	1.99 (1.92-2.07)	<0.0001	1.37 (1.32-1.44)	<0.0001
Self- Pay	4.03 (3.82-4.26)	<0.0001	2.04 (1.93-2.17)	<0.0001
Others	4.81 (4.56-5.06)	<0.0001	2.89 (2.74-3.05)	<0.0001
Hospital Teaching Status				
Rural	Ref			
Urban non-teaching	1.19 (1.09-1.30)	0.244		
Urban teaching	2.82 (2.57-3.10)	<0.0001		

However, the following were associated with reduced odds of attempted suicide and self-inflicted injury: age group ≥65 years (OR 0.16 (0.15-0.17)), 40-64 years (OR 0.25 (0.24-0.26)), and 25-39 years (OR 0.54 (0.52-0.56)) compared to 18-24 years; and blacks (OR 0.84 (0.79-0.90)) compared to whites (Table 2). Mental health disorders (OR 0.55 (0.54-0.58)) and substance use disorder (OR 0.94 (0.89-0.99)) were associated with decreased odds of attempted suicide and self-inflicted injury (Table 2).

## Discussion

The prevalence of attempted suicide and self-inflicted injury was higher in white males age 40-64 years old. This differs from other studies that found higher rates among youth age 15-19 years old and age 21-34 years old who visited the Emergency Department (ED) [6,19-21]. However, these studies identified the risk difference of completed suicide highest among adults age 45-64 years. The reason we might have found a higher number of hospitalizations for the age group 40-64 years is that this range is of a higher risk for suicide completions warranting more critical inpatient care for this age group compared to others. We also observed that whites over other races were found to have attempted suicide and self-inflicted injury which is consistent with results from other studies [22,23]. Many studies found that attempted suicide and self-inflicted injury were higher in females [6,21], but our study found that there were more suicide attempts in males. This finding is also supported by another study conducted in Europe [24]. Although generally, research shows that women attempt suicide two to three times more often than men while men are four times more likely to complete suicide [19,25]. The more critical inpatient care needs mentioned earlier for ages 40-64 years may also be responsible for the difference in gender prevalence. Studies have shown that there is a higher fatality rate reaching a peak of 55.2% in males 65 years and older [2]. Temporally, our study continued to show a consistent rise in prevalence for both genders, which suggests that treatment and intervention should be targeted for both genders.

This study showed a total of about 249,845 admissions for attempted suicide and self-inflicted injury over the five-year period. This is a steady increase in the prevalence rate of about 168-200 per 100,000 hospitalization rates with a much higher and steeper rise in prevalence among the male population with a statistically significant difference; however, both genders did show a positive trend. This prevalence rate is comparable with a similar study which reported prevalence from 163.1 to 173.8 per 100,000 admissions from 2006 to 2013 [2]. This is contrary to what some other studies found in which non-fatal suicide-related injuries between 2006 and 2013 accounted for a higher female to male ratio (1.29:1) [2].

In our analysis, we generated a seasonal pattern of attempted suicide hospitalization with peak periods in late summer (July and August) over the study period. However, different studies have found suicides occurring in late spring [26,27] and others have found a second peak during summer [28,29]. A study found that winter months from October to January had the lowest rates; however, in our study, January to March seems to be the lowest prevalence for attempted suicide and self-inflicted injury [26].

We found that attempted suicide and self-inflicted injury was more likely time to take place during the weekends compared to weekdays. There was also a significant difference in the higher rate of attempt during the weekend compared to the weekday (p<0.05). The NIS data only distinguishes between weekend and weekday but does not give specific days of the week. A study reported that the highest frequencies for attempted suicide and self-inflicted injury were on Monday and Tuesday [26].

Studies have consistently shown that patients with attempted suicide and self-inflicted injury are likely to have a prior history of mental illnesses including depression, anxiety, psychosis, and antisocial personality disorders [6]. The prevalence of these mental conditions increases with the number of patients with attempted suicide and self-inflicted injury. Our study showed reduced odds of mental disorders in patients who attempted suicide and self-inflicted injury. The reason for this finding in our study is unclear. However, it is estimated that 90% of those who die by suicide have a diagnosed mental illness, and most often unrecognized or untreated depression [20]. In our study, 8.2% of patients had depressive disorder. Doganay et al. found that those who had depressive disorder had suicidal tendencies in the spring and summer months, which is consistent with findings in our study [30].

A limitation of this study is the source of the database. The NIS database is an administrative database used primarily for billing and is prone to coding errors. E-codes were used to identify injuries related to attempted suicide, which are not typically required when billing trauma patients. In addition, the collection of E-codes varies across states, as some states require the collection of E-codes and some other states do not require it. It can be underreported in some cases, making the reporting less accurate. Another limitation is the inability to distinguish between a self-inflicted injury that was due to a suicidal or non-suicidal intention.

The strength of this study is the availability of a large sample size and a longitudinal analytical database with clinical and demographic data across the US. This large database increases study power and generalizability of findings.

## Conclusions

Awareness of seasonal and epidemiological trends of attempted suicide and self-inflicted injury is a very important step to develop effective strategies to prevent suicide. The prevalence of attempted suicide is steadily rising 168-200 per 100,000 hospitalization rates per year affecting both males and females. Our study showed seasonal and weekly patterns of suicide attempts with peak periods during late summer and weekends. A better understanding of the most commonly affected population demographic is important when evaluating options for treatment and intervention.

## Appendices

**Table 3:** ICD-9 codes used for mental health and substance use disorders ICD-9, International Classification of Diseases-9

Diagnosis	ICD-9 codes
Alcohol	303.00, 303.01, 303.02, 303.03, 303.90, 303.91, 303.92, 303.93, 305.00, 305.01, 305.02, 305.03, 980.0, 980.1, 980.2, 980.3, 980.8, 980.9
Marijuana	304.30, 304.31, 304.33, 305.20, 305.21, 305.22, 305.23
Sedative-hypnotic	304.10, 304.11, 304.13, 305.40, 305.41, 305.42, 305.43, 967.0, 967.1, 967.2, 967.3, 967.4, 967.5, 967.6, 967.8, 967.9, 969.4, E851, E852.0, E852.1, E852.2, E852.3, E852.4, E852.5, E852.8, E852.9, , E853.0, E853.1, E853.2, E853.8, E853.9, E937.0, E937.1, E937.2, E937.3, E937.4, E937.4, E937.5, E937.6, E937.7, E937.8, E937.9
Cocaine	304.20, 304.22, 304.23, 305.60, 305.61, 305.62, 305.63, 970.81, 970.89
Stimulant	304.00, 304.41, 304.42, 304.43, 305.70, 305.71, 305.72, 305.73, 969.6, 970.0, 970.1, 970.9, E854.2, E854.3, E854.9
Hallucinogen	304.50, 304.51, 304.52, 304.53, 305.30, 305.31, 305.32, 305.53, E854.1, E855.5, E855.6, E855.8, E855.9
Others	304.60, 304.61, 304.62, 304.63, 304.70, 304.71, 304.72, 304.73, 304.80, 304.81, 304.83, 304.90, 304.91, 304.92, 304.93, 305.80, 305.81, 305.82, 305.83, 305.90, 305.91, 305.92, 305.93, 969.0, E939.0, E939.1, E939.2, E939.2, E939.3, E939.4, E939.5, E939.6, E939.6, E939.7, E939.8, E939.9
Mood disorder	293.83, 296.00, 296.01, 296.02, 296.03, 296.04, 296.05, 296.06, 296.10, 296.11, 296.12, 296.13, 296.14, 296.15, 296.16, 296.20, 296.21, 296.22, 296.23, 296.24, 296.25, 296.26, 296.30, 296.31, 296.32, 296.33, 296.34, 296.35, 296.36, 296.40, 296.41, 296.42, 296.43, 296.44, 296.45, 296.46, 296.50, 296.51, 296.52, 296.53, 296.54, 296.55, 296.56, 296.60, 296.61, 296.62, 296.63, 296.64, 296.65, 296.66, 296.7, 296.80, 296.81, 296.82, 296.89, 296.90, 296.99, 300.4, 311
Anxiety	293.84, 300.00, 300.01, 300.02, 300.09, 300.10, 300.20, 300.21, 300.22, 300.23, 300.29, 300.3, 300.5, 300.89, 300.9, 308.0, 308.1, 308.2, 308.3, 308.4, 308.9, 309.81, 313.0, 313.1, 313.21, 313.22, 313.3, 313.82, 313.83
Psychosis	297.0, 297.1, 297.2, 297.3, 297.8, 297.9, 298.0, 298.1, 298.2, 298.3, 298.4, 298.8
Depression disorder	311, 296.82, 296.20, 296.21, 296.22, 296.23, 296.24, 296.25, 296.26, 296.30, 296.31, 296.32, 296.33, 296.34, 296.35, 296.36, 296.50, 296.51, 296.52, 296.53, 296.54, 296.55, 296.56
